# Impact of Sarcopenia on Mortality in Patients Undergoing TAVI: A Follow-Up Study

**DOI:** 10.3390/jcm14093182

**Published:** 2025-05-04

**Authors:** Víctor Navas Moreno, Fernando Sebastián-Valles, Elena Carrillo López, Alicia Justel Enríquez, Carolina Sager La Ganga, Miguel Antonio Sampedro-Núñez, Víctor Rodríguez Laval, Nuria Sánchez de la Blanca, Álvaro Montes Muñiz, Fernando Alfonso Manterola, Luis Jesús Jiménez-Borreguero, Mónica Marazuela

**Affiliations:** 1 Department of Endocrinology and Nutrition, Hospital Universitario de La Princesa, Universidad Autónoma de Madrid, 28028 Madrid, Spain; victor.navas@salud.madrid.org (V.N.M.); fernando.sebastian@estudiante.uam.es (F.S.-V.); nsdelablanca.96@gmail.com (N.S.d.l.B.); 2 Instituto de Investigación Sanitaria Princesa (IIS-IP), 28028 Madrid, Spain; 3 Department of Radiology, Hospital Universitario de la Princesa, 28028 Madrid, Spain; vrlaval@gmail.com; 4 Department of Cardiology, Hospital Universitario de la Princesa, 28028 Madrid, Spainljborreguero@gmail.com (L.J.J.-B.)

**Keywords:** sarcopenia, TAVI, survival, body composition, computed tomography

## Abstract

**Objective:** The use of transcatheter aortic valve implantation (TAVI) has expanded in patients with severe aortic stenosis who are deemed inoperable. However, sarcopenia may be a determining factor in their survival. The aim of our study is to assess the impact of sarcopenia, evaluated by computed tomography (CT), on mortality in this patient population. **Methods:** Patients with severe aortic stenosis undergoing follow-up after TAVI at Hospital Universitario de la Princesa were recruited. Body composition was analyzed using routine CT scans and open-source software. Survival analysis was performed, and correlations between body composition parameters at the T12 and L3 vertebral levels were assessed. **Results:** Our sample comprised 97 subjects. Time to mortality was associated with diabetes mellitus (*p* = 0.050), atrial fibrillation (*p* = 0.02), and respiratory disease (*p* = 0.03). Interestingly, sarcopenia (*p* = 0.039) and normal-density muscle area (*p* = 0.025) were also associated with time to mortality, with the association between sarcopenia and time to mortality becoming stronger after adjusting for covariates (*p* < 0.001). The correlation between different body composition parameters at the T12 and L3 vertebral levels was substantial and statistically significant. **Conclusions:** The use of CT to assess sarcopenia in patients with severe aortic stenosis undergoing TAVI is highly valuable and can predict time to mortality. Sarcopenia should be considered as a relevant parameter in the comprehensive evaluation of these patients.

## 1. Introduction

Transcatheter aortic valve implantation (TAVI) has emerged as a groundbreaking treatment for patients with severe aortic stenosis (AS) who are deemed high-risk or ineligible for conventional surgical aortic valve replacement [1,2]. This minimally invasive procedure has significantly improved survival rates and quality of life in elderly and frail patients [3]. However, despite its benefits, post-procedural outcomes remain highly variable, influenced by multiple factors including comorbidities, frailty, and nutritional status [4,5].

In recent years, body composition analysis has gained increasing recognition as a key element in assessing patient prognosis and guiding clinical decision-making [6]. Traditional anthropometric measures such as body mass index (BMI) have limitations in accurately reflecting a patient’s nutritional and functional status [7]. In contrast, advanced imaging techniques, particularly computed tomography (CT), offer a more precise and detailed evaluation of body composition [6]. By analyzing parameters such as skeletal muscle mass, visceral and subcutaneous fat distribution, and muscle quality, CT-based assessments can provide valuable insights into sarcopenia, cachexia, and fat excess [8,9]—conditions that have been linked to poorer post-TAVI outcomes [10].

Sarcopenia, characterized by the progressive loss of skeletal muscle mass and function, has been associated with increased mortality, prolonged hospital stays, and higher rates of complications in cardiovascular diseases [11]. Given that CT imaging is routinely performed for procedural planning in TAVI candidates, leveraging this modality for body composition assessment presents a unique opportunity to enhance risk stratification without additional diagnostic burden.

Quality of life (QoL) is a central outcome in patients undergoing TAVI for severe aortic stenosis. Unlike chronic heart failure, particularly heart failure with reduced eject fraction (HFrEF), which is often irreversible, aortic stenosis represents a potentially reversible cause of heart failure, typically presenting with heart failure with preserved ejection fraction (HFpEF) features [12]. This distinction highlights the potential for significant improvement in QoL after TAVI and supports the relevance of evaluating factors like sarcopenia, which directly impact functional recovery.

This study aims to evaluate body composition using CT imaging in patients undergoing TAVI and investigate its potential role in predicting clinical outcomes, particularly mortality and time to death. As a secondary objective, the correlation between body composition parameters at the lumbar and thoracic levels will be assessed.

## 2. Materials and Methods

### 2.1. Population

The study includes patients with severe aortic stenosis who underwent TAVI between 2017 and 2019 at Hospital Universitario de la Princesa (Interventional Cardiology Department). Patients were followed up until December 2024.

Inclusion criteria were as follows:

1. Men and women of any age who received the prosthesis.
2. Availability of thoracic and/or abdominal CT images suitable for sarcopenia assessment.
3. CT imaging performed prior to TAVI placement.

Exclusion criteria were as follows:

4. Patients with imaging studies who ultimately did not undergo TAVI.
5. Patients with unavailable imaging studies.
6. Patients with available imaging studies in which sarcopenia assessment was not feasible.
7. Patients without clinical follow-up.

Ethics approval was granted by the Internal Ethics Committee of our institution, La Princesa University Hospital, and the study was conducted in accordance with the Declaration of Helsinki. The internal ethics committee’s code was 5618.

### 2.2. TAVI Performance

Transcatheter aortic valve implantation (TAVI) procedures were performed according to the clinical criteria of the Cardiology Department (patients with severe aortic stenosis who are not candidates for surgery).

#### Pre-Procedural Planning and TAVI Procedure

Patients with suspected severe aortic stenosis were evaluated by transthoracic echocardiography (TTE). Valve morphology, mean gradient (MG), and aortic valve area (AVA) were assessed and severe aortic stenosis was diagnosed in cases with MG > 40 mmHg and AVA < 1 cm^2^. Low-flow, low-gradient aortic stenosis cases were further confirmed with CT calcium scoring as recommended in current valvular heart disease guidelines [13]. Cases were presented in Heart Team and those deemed unsuitable for surgical valve replacement were accepted for TAVI. Pre-procedural planning included a coronary catheterization and CT angiography to assess the aortic root anatomy and access route.

The procedure was performed under conscious sedation and the vascular access was femoral, with a secondary access in the contralateral femoral artery or radial artery. A self-expanding prosthetic valve was deployed, guided by fluoroscopy and TTE. Vascular closure devices were used to achieve hemostasis. Post-procedural care took place in a cardiac intensive care unit.

Follow-up after the procedure was carried out according to clinical criteria including annual TTE to assess prosthetic function and LV structural and functional changes [14].

### 2.3. Clinical Data

Biochemical and fundamental anthropometric data were extracted from electronic health records. We collected data on anthropometry, comorbidities, and biochemical parameters related to nutrition, as well as information on TAVI placement, including catheter-related complications, ischemic heart disease, arrhythmias, cardiac rupture, cerebrovascular accident, and all-cause mortality.

### 2.4. CT Image Analysis

Body composition was evaluated by analyzing CT images acquired as part of routine clinical practice before TAVI placement. An experienced radiologist selected high-quality CT images of patients at the T12 and L3 level. Images with high contrast, absence of artifacts, and visible surgical interventions were excluded. It was confirmed that there were no medications that could interfere with the CT scan image prior to the procedure. CT image processing was performed using NIH ImageJ version 2.3.0 (National Institutes of Health, Bethesda, Maryland, USA) [15], following the protocol described by our group in previous work [8]. Subsequently, area subtraction was executed using R version 4.0.3 (R Foundation for Statistical Computing, Vienna, Austria) [16].

The following body composition measurements were obtained: total body area, visceral fat tissue (VFA; HU = −190, −30), subcutaneous fat area (SFA; HU = −190, −30), intermuscular fat area (IMFA; HU = −190, −30), total fat area (TFA; HU = −190, −30), very low-density muscle area (VLDMA; HU = −29, −1), low-density muscle area (LDMA; HU = 0, 34), normal-density muscle area (NDMA; HU = 35, 100), high-density muscle area (HDMA; HU = 101, 150), very high-density muscle area (VHDMA; HU = 151, 199), and total muscle area (TMA; HU = −29, 199) [17]. After the measurement step, correlation between both analyses was tested using Spearman’s rho test. Subsequently, the data were normalized by dividing them by the square of patients’ height in meters. Finally, the mean of both measurements was obtained.

In this study, sarcopenia was defined as follows: low muscle mass (L3 skeletal muscle index (SMI) ≤ 50.2 cm^2^/m^2^ in males and ≤34.9 cm^2^/m^2^ in females) [18,19].

### 2.5. Statistical Analysis

Categorical variables were expressed as frequencies and percentages, and compared using the chi-square test or Fisher’s exact test, as appropriate. Continuous variables were presented as mean ± standard deviation (SD) or median with interquartile range (IQR), depending on their distribution. Normality was assessed using the Kolmogorov–Smirnov test and visual inspection of histograms. Group comparisons for continuous variables were performed using the Student’s t-test for normally distributed data, or the Mann–Whitney U test for non-normally distributed data. A *p*-value < 0.05 was considered statistically significant.

A correlation matrix was constructed to identify associations between clinical parameters and body composition markers at the T12 vertebrae and L3 vertebrae (positive rho values indicated variation in the same direction, while negative rho values indicated variation in opposite directions).

A survival time analysis was performed, considering the time until death for deceased patients and the time until the last medical visit for survivors which was plotted using a Kaplan-Meier curve. Subsequently, a Cox proportional hazards model was conducted to assess the association between various clinical, analytical, and body composition variables with less survival time. Variables associated with mortality and/or sarcopenia were included in the multivariable analysis. In order to limit overfitting, we followed the recommendation by Peduzzi et al. regarding the number of events per variable [20]. Variable selection was based on clinical relevance and statistical significance in the bivariate analysis, following Rothman’s criteria [21]. These variables were chronic respiratory disease, pacemaker requirement, DM, and atrial fibrillation; sex and age were also included. Finally, we analyzed whether sarcopenia was independently associated with survival time, regardless of these other related variables.

For all statistical analyses, STATA 18.0 BE-Basic Edition (Lakeway Drive, College Station, TX, USA), Graphpad Prism version 9 (Boston, MA, USA), and R version 4.0.3 were employed. A *p*-value < 0.05 was considered statistically significant.

## 3. Results

### 3.1. Sample Characteristics

The final sample consisted of 97 patients (Table 1). The median age at the time of TAVI was 85.4 years (83.6–87.9 y), and 38.1% of the patients were male. By the end of the follow-up period, 53.6% of the patients had died. Diabetes mellitus was present in 33%, dyslipidemia in 72.2%, hypertension in 85.6%, ischemic heart disease in 31.3%, atrial fibrillation in 36.1%, and respiratory disease in 16.5% of the cases. Based on body composition radiomic parameters, sarcopenia was diagnosed in 60% of the subjects.

### 3.2. Clinical and Biochemical Parameters, and Complications Associated with Time to Mortality

In the Cox proportional hazard models, time to mortality was found to be associated with a history of DM (HR 1.74, 95% CI [1.01–3.02], *p* = 0.050), atrial fibrillation (HR 1.90, 95% CI [1.09–3.32], *p* = 0.02), and respiratory disease (HR 2.00, 95% CI [1.05–3.83], *p* = 0.03). Regarding complications, it was associated with the need for a pacemaker (HR 2.09, 95% CI [1.15–3.81], *p* = 0.022) and post-intervention acute kidney failure (HR 1.71, 95% CI [1.08–2.69]; *p* = 0.021).

No association was observed between time to mortality and other medical history factors such as ischemic heart disease, stroke, or active smoking. Likewise, no statistically significant association was found with other complications, such as those related to vascular access or ischemic heart disease, probably due to the low number of patients presenting these conditions.

### 3.3. Clinical and Biochemical Parameters, and Complications Associated with Sarcopenia

Sarcopenia was defined according to previous studies (as described in the Section 2) and was analyzed univariably in relation to other variables. An inverse association was found with BMI (coefficient −2.9, 95% CI [−5.47–−0.27], *p* = 0.031), and a positive association with DM (Pearson chi^2^ = 4.13, *p* = 0.042).

### 3.4. Body Composition Parameters, Including Sarcopenia, Are Associated with Mortality

**Figure 1:**
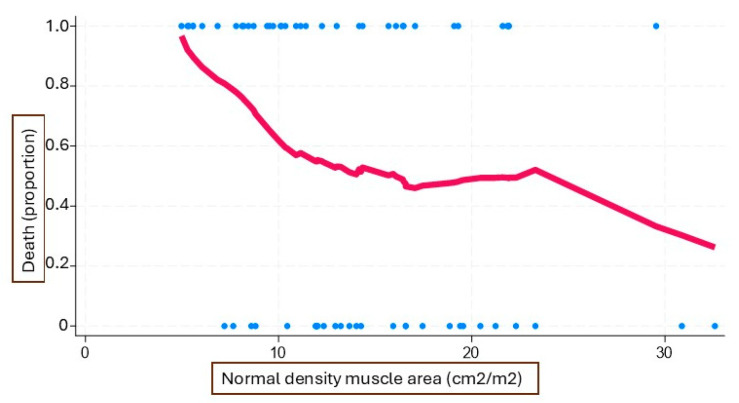
LOESS plot of the relationship between normal-density muscle area and mortality. The x-axis represents the normal-density muscle area, while the y-axis shows the proportion of deceased patients. Blue dots represent individual patient observations, while the red line indicates the LOESS-smoothed trend, illustrating the overall pattern of association without assuming a predefined functional form. As shown in the figure, the relationship between normal-density muscle and mortality indicates that death is more likely when the normal-density muscle area is less than 10 cm^2^/m^2^, plateaus between 10 and 20 cm^2^/m^2^, and is highly unlikely when it exceeds 20 cm^2^/m^2^.

In the univariate analysis, sarcopenia was associated with mortality (chi^2^ = 4.24, *p* = 0.039); in this context, normal-density muscle at the L3 level was associated with a decreased risk of mortality (HR 0.93, 95% CI [0.87–0.99], *p* = 0.025). This parameter was highly useful for phenotyping mortality risk (sigmoid curve with cutoff points of <10 cm^2^/m^2^, 10–20 cm^2^/m^2^, and > 20 cm^2^/m^2^) (Figure 1). It shows an acceptable correlation with the classical sarcopenia measurement proposed by Prado et al. [22,23].

Finally, a multivariable analysis was performed (Table 2), adjusting the results by including variables associated with time to mortality and/or sarcopenia: BMI, respiratory disease, atrial fibrillation, diabetes mellitus, and need for a pacemaker. Age and sex were also included due to their potential influence on sarcopenia. The results were robust, with sarcopenia representing a clear increase in risk (HR 3.30, 95% CI [1.33–8.19], *p* < 0.001).

**Figure 2:**
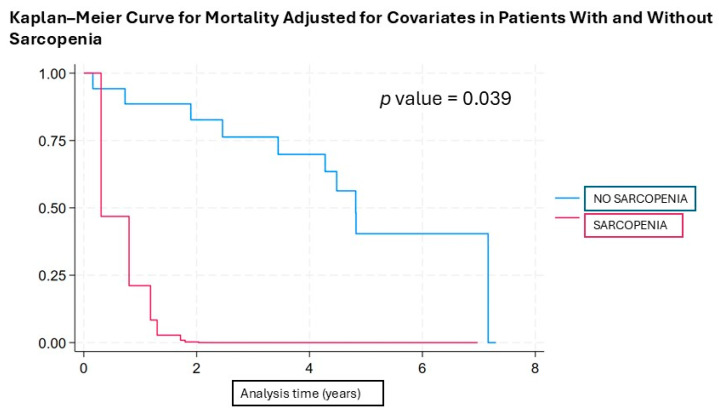
Kaplan–Meier survival curve by subgroup (A—Blue: No Sarcopenia; B—Red: Sarcopenia) adjusted for age, sex, BMI, respiratory disease, diabetes mellitus, atrial fibrillation, and need for a pacemaker.

These adjusted results are illustrated in Figure 2 with a survival curve, demonstrating that survival time is shorter in individuals with sarcopenia compared to those with normal muscle mass.

### 3.5. Correlation of Body Composition Parameters Between the T12 and L3 Vertebral Levels

**Figure 3:**
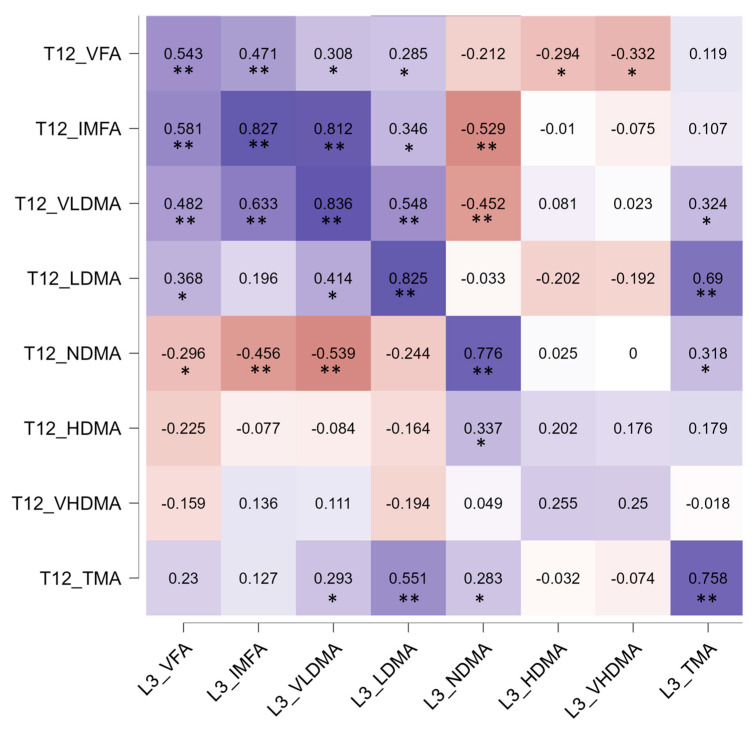
Correlation matrix of body composition parameters at T12 and L3 measured by CT. Values represent the Spearman’s rank correlation coefficient, rho (ρ) between body composition measured at T12 and L3; both CT scans were performed on the same date. * indicates *p*-value < 0.05, and ** indicates *p*-value < 0.001. Significant negative correlations are shown in orange and significant positive correlations in blue. Color intensity increases with the magnitude of correlation. White-colored cells indicate a nonsignificant correlation. T12 and L3 preceding the composition parameter indicate whether it is at the level of the T12 or L3 vertebra. HDMA, high-density muscle area; IMFA, intermuscular fat area; LDMA, low-density muscle area; NMDA, normal-density muscle area; TMA, total muscle area; VFA, visceral fat area; VHDMA, very high-density muscle area; VLDMA, very low-density muscle area.

A Spearman correlation matrix was performed to study the association between body composition parameters at the T12 and L3 vertebral levels (Figure 3).

The main body composition parameters showed a statistically significant positive correlation: NDMA (ρ = 0.707, *p* < 0.001), LDMA (ρ = 0.750, *p* < 0.001), VLDMA (ρ = 0.854, *p* < 0.001), intramuscular fat (ρ = 0.865, *p* < 0.001), TMA (ρ = 0.738, *p* < 0.001), and VFA (ρ = 0.678, *p* < 0.001). There was no correlation with high-density and very high-density muscle, likely because the amount of these muscle types was very small in the sample. Subcutaneous fat was not analyzed, as it was truncated in several imaging studies at that level.

## 4. Discussion

This study aimed to evaluate the impact of sarcopenia, assessed by CT at the time of TAVI, on mortality in patients with severe aortic stenosis. Our results showed that sarcopenia is independently associated with mortality, regardless of other covariates.

The use of TAVI for inoperable patients with severe aortic stenosis has represented a paradigm shift in recent decades in terms of improving quality of life [24]. However, it is a procedure not without serious and potentially life-threatening complications [5,25], which may be influenced by the body composition of these patients [10,26]. Therefore, it is essential to have tools to assess these parameters and stratify risk in this population.

In our study, we observed an association between time to mortality and comorbidities such as diabetes mellitus, atrial fibrillation, and respiratory disease, which is in line with previous studies. Atrial fibrillation has been associated with an increased risk of bleeding in previous studies, and the latter with a higher risk of mortality within 30 days post-intervention [27]. DM has been associated with other complications, such as acute kidney injury and the need for dialysis [28], and this finding is of particular interest considering previous research suggesting a similar link. Notably, one study reported that sodium-glucose co-transporter 2 inhibitors (SGLT2i) were associated with reduced mortality and more favorable cardiac remodeling [29]. Furthermore, chronic respiratory disease has been associated with a risk of both short-term and long-term mortality in previous studies [30]. Regarding complications associated with mortality, our study identified the need for a pacemaker and acute kidney failure, consistent with findings in previous studies [25,31].

Sarcopenia is defined as the loss of muscle mass, muscle strength, and/or muscle function in the context of disease or aging, leading to malnutrition [32]. The importance of this condition lies in its association with mortality across a wide range of diseases, increased hospital length of stay, readmissions, and healthcare costs [33,34,35,36]. In patients undergoing TAVI, the worsening of outcomes has been observed in those with sarcopenia, including an increase in complications and even mortality [26,37], although in some studies, this was only observed in males [10,38] and in another this was only observed in a subgroup of females [39]. This is likely due to the heterogeneity of the studies, arising from the lack of international consensus on the definition of sarcopenia criteria and the methods used in each study. However, in our study, sarcopenia was associated with time to all-cause mortality in the univariable model, indicating a significant increase in risk. This risk was further amplified after adjusting for variables associated with mortality and sarcopenia itself in the multivariable model and in the Kaplan–Meier survival, which also included age and sex.

In our study, in addition to sarcopenia, we examined the association between time to all-cause mortality and the amount of normal-density muscle, finding a significant correlation. No association was observed with other muscle densities, likely due to the limited amount of high and very high-density muscle in our cohort. This was also confirmed in the work of Tokuda et al. [40], where muscle density was associated with post-procedural complications. Interestingly, in our study, when the area of normodense muscle was divided into tertiles, mortality was higher in those with a lower amount, consistent with lower mortality in those with the highest tertile, with the middle tertile representing a plateau zone.

Our findings should also be interpreted in the context of evolving imaging modalities. While transthoracic echocardiography (TTE) remains the standard for assessing structural and functional changes post-TAVI, advanced echocardiographic techniques offer a more detailed evaluation of myocardial mechanics and concomitant valvular disease, potentially refining outcome prediction and patient selection [14]. Given the multifactorial nature of this process, the approach to management should also be multifaceted. Optimizing nutrition and incorporating resistance training exercises are likely to be important in these patients. However, to date, there is a lack of studies that thoroughly investigate these aspects.

The coexistence of sarcopenia, heart failure, and severe aortic stenosis underscores the multifactorial nature of adverse outcomes in this population. As shown in the SICA-HF study [41], sarcopenia is common in heart failure and may compound the effects of cardiac dysfunction and valve disease. Understanding this interplay is key to improving prognosis and guiding management strategies.

Taking advantage of the availability of thoracic cuts in the CT scans of the patients in our cohort, we decided to expand the study and examine whether there was a correlation at the T12 and L3 levels. Sarcopenia at the T12 level was not studied, as there are no established cutoff values to define it at this level. In a previous study from our group [8], a correlation was demonstrated between body composition parameters measured by bioimpedance and the T12 level. In other studies, a correlation has been demonstrated between T12 and lumbar cuts in CT scans [42,43], as seen in our study. This suggests that thoracic cuts could be used for body composition analysis in the population with valvular heart disease.

## 5. Limitations

This study has several limitations. Being unicentric limits the external validation of the results, although most previous TAVI studies are also unicentric. We did not have subcutaneous fat cuts available, which prevented us from conducting analysis of subcutaneous fat and total fat; however, the primary objective of our study was to evaluate muscle mass. We believe it would be very useful to assess these body composition parameters in conjunction with frailty questionnaires and scales, but we did not have that data in our cohort. Another limitation of our study is the lack of data on key aortic stenosis parameters (mean gradient, peak velocity, aortic valve area), as well as left ventricular ejection fraction and the presence of low-flow low-gradient aortic stenosis. Additionally, the observational nature of this study limits the ability to draw causal inferences, restricting our conclusions to hypothesis-generating findings.

## 6. Conclusions

In summary, the assessment of body composition using CT in patients with severe aortic stenosis awaiting TAVI may provide valuable information regarding the risk of complications and mortality post-procedure. The evaluation of sarcopenia, along with other comorbidities, could be considered as part of the decision-making process for frail patients. Additionally, our study suggests that the T12 cut could be useful for obtaining these parameters. The potential role of nutritional and exercise interventions in preventing or managing sarcopenia in patients with severe aortic stenosis warrants further exploration.

## Figures and Tables

**Table 1:** Sample characteristics, including patients stratified by mortality status. Comparison of baseline data between deceased and non-deceased groups.

	Median	Non Death	Death	*p* Value *
n	97	45 (46.4%)	52 (53.6%)	
Sex: men	37 (38.1%)	17 (37.8%)	20 (38.5%)	0.945
Age (y)	85.4 (83.6–87.9)	84.9 (82.2–86.1)	86.2 (84.0–89.4)	0.013
BMI (kg/m^2^)	25.7 (24.3–29.1)	25.4 (24.3–29.0)	25.8 (24.2–30.5)	0.979
Smoker	36 (37.1%)	16 (35.6%)	20 (38.5%)	0.768
DM	32 (33%)	9 (20%)	23 (44.2%)	0.012
Dyslipemia	70 (72.2%)	32 (71.1%)	38 (73%)	0.830
Hypertension	83 (85.6%)	38 (84.4%	45 (86.5%)	0.771
IHD	31 (31.3%)	12 (26.7%)	19 (36.5%)	0.301
Atrial fibrilation	35 (36.1%)	12 (26.7%)	23 (44.3%)	0.074
Respiratory	16 (16.5%)	4 (8.9%)	12 (23.1%)	0.062
Sarcopenia	39/65 (60%)	13 (46.4%)	26 (70.3%)	0.054

BMI: body mass index. DM: Diabetes Mellitus. IHD: ischemic heart disease. Respiratory: chronic respiratory pathology. * A *p*-value below 0.05 is considered statistically significant.

**Table 2:** Multivariable analysis of time to mortality. The model was adjusted for covariates associated with time to mortality and/or sarcopenia.

	Hazard Ratio [CI]	*p* Value *
Sarcopenia	3.30 [1.33–8.19]	0.01
BMI	1.00 [0.92–1.08]	0.97
Sex	0.64 [0.26–1.56]	0.32
Age	0.97 [0.88–1.07]	0.53
Respiratory	2.42 [0.88–6.62]	0.09
Peacemaker	4.42 [2.08–9.37]	<0.001
DM	2.18 [1.01–4.73]	0.04
Atrial fibrillation	1.71 [0.77–3.76]	0.19

BMI: body mass index. DM: Diabetes Mellitus. Respiratory: chronic respiratory pathology. * A *p*-value below 0.05 is considered statistically significant.

## Data Availability

V.N.M. and M.M. are the guarantors of this work and, as such, had full access to all of the data in this study and take responsibility for the integrity of the data and the accuracy of the data analysis.

## Abbreviations

The following abbreviations are used in this manuscript:

MDPI	Multidisciplinary Digital Publishing Institute
DOAJ	Directory of open access journals
TLA	Three letter acronym
LD	Linear dichroism
